# Institutional Questions

**Published:** 1899-11-04

**Authors:** 


### INSTITUTIONAL QUESTIONS.

### The Lunacy Laws.

"Interested" writes: Can you explain to me the working of the Lunacy Laws respecting the detention of persons of unsound mind in private houses; and what is the precise meaning of "licensed" or "unlicensed" houses in this connection?

*** You will find the whole of the law applying to private lunatic patients in the Act passed in 1890, 53 Vict., c. 5. Private patients "cannot be received in an asylum, hospital, or licensed house, nor can a single patient be received in a private house without a reception order made by a County Court judge, a magistrate, or a justice specially appointed; but this does not apply in the case of a lunatic so found by inquisition, or where an 'emergency order' has been obtained. A reception order is made upon a private application, by petition, with a statement of particulars, accompanied by two medical certificates under the hands of two medical practitioners." A single patient may be received on a reception or a temporary urgency order without the necessity for the house being registered as an asylum, and the Commissioners in Lunacy have power, under special circumstances, to allow two or more patients to be received in an unlicensed house. Licensed houses are private asylums maintained by private individuals at their own risk and for their own profit.

### Homes for Incurables.

A correspondent asks for the names of homes for incurables in the northern counties, particularly in Yorkshire.

*** An excellent institution for cancer and incurables is St. Catherine's Home, St. Mary's Road, Bradford, for women patients only. There is at Harrogate the Yorkshire Home for Chronic and Incurable Diseases; address Secretary, 13, Ash Grove, Harrogate. At Paisley, the Gleniffer Home for Treatment of Diseases Considered Incurable, Corsebar Road

At Manchester there is the Northern Counties Hospital for Incurables, which has branch homes attached. A full list of such institutions will be found in "Hospitals and Charities."

### Staining for Floors.

"Matron" asks: Can you give me a good recipe for making a floor staining at less cost than is incurred by buying it by the quart at an ironmonger's?

*** The following will be found a very good method of staining and polishing floors at small cost. Pour boiling water on permanganate of potash crystals, in the proportion of one ounce of permanganate to one pint of water. Paint the mixture on the boards with a wide brush till the required depth of colour is obtained. When quite dry rub in the following polish: One and a half pounds of beeswax, five ounces resin, one pint of turpentine. Melt the beeswax and resin together and while liquid stir in the turpentine, taking care to do this at a distance from the fire to avoid accident. The secret of getting a good appearance with floors treated in this manner is to see that the polish is rubbed in until the surface is quite bright and no trace of stickiness remains.

